# Protective effects of total fraction of avocado/soybean unsaponifiables on the structural changes in experimental dog osteoarthritis: inhibition of nitric oxide synthase and matrix metalloproteinase-13

**DOI:** 10.1186/ar2649

**Published:** 2009-03-16

**Authors:** Christelle Boileau, Johanne Martel-Pelletier, Judith Caron, Philippe Msika, Georges B Guillou, Caroline Baudouin, Jean-Pierre Pelletier

**Affiliations:** 1 Osteoarthritis Research Unit, University of Montreal Hospital Centre (CRCHUM), Notre-Dame Hospital, Sherbrooke Street East, Montreal, Quebec H2L 4M1, Canada; 2 Laboratoires Expanscience, Avenue de l'Arche, 92419 Courbevoie Cedex, France

## Abstract

**Introduction:**

The aims of this study were, first, to investigate the *in vivo *effects of treatment with avocado/soybean unsaponifiables on the development of osteoarthritic structural changes in the anterior cruciate ligament dog model and, second, to explore their mode of action.

**Methods:**

Osteoarthritis was induced by anterior cruciate ligament transection of the right knee in crossbred dogs. There were two treatment groups (n = 8 dogs/group), in which the animals received either placebo or avocado/soybean unsaponifiables (10 mg/kg per day), which were given orally for the entire duration of the study (8 weeks). We conducted macroscopic and histomorphological analyses of cartilage and subchondral bone of the femoral condyles and/or tibial plateaus. We also conducted immunohistochemical analyses in cartilage for the following antigens: inducible nitric oxide synthase, matrix metalloproteinase (MMP)-1, MMP-13, a disintegrin and metalloproteinase domain with thrombospondin motifs (ADAMTS)4 and ADAMTS5.

**Results:**

The size of macroscopic lesions on the tibial plateaus was decreased (*P *= 0.04) in dogs treated with the avocado/soybean unsaponifiables. Histologically, in these animals the severity of cartilage lesions on both tibial plateaus and femoral condyles, and the cellular infiltration in synovium were significantly decreased (*P *= 0.0002 and *P *= 0.04, respectively). Treatment with avocado/soybean unsaponifiables also reduced loss of subchondral bone volume (*P *< 0.05) and calcified cartilage thickness (*P *= 0.01) compared with placebo. Immunohistochemical analysis of cartilage revealed that avocado/soybean unsaponifiables significantly reduced the level of inducible nitric oxide synthase (*P *< 0.05) and MMP-13 (*P *= 0.01) in cartilage.

**Conclusions:**

This study demonstrates that treatment with avocado/soybean unsaponifiables can reduce the development of early osteoarthritic cartilage and subchondral bone lesions in the anterior cruciate ligament dog model of osteoarthritis. This effect appears to be mediated through the inhibition of inducible nitric oxide synthase and MMP-13, which are key mediators of the structural changes that take place in osteoarthritis.

## Introduction

Treatment of osteoarthritis (OA) is becoming a major medical issue, with aging of the world's population. This disease is by far the most common musculoskeletal disorder, and it is responsible for a significant portion of the financial costs related to treatment of arthritic conditions. With the predicted increase in incidence of OA in coming decades, the costs related to this disease are becoming a serious concern. More people are surviving major medical problems such as cardiovascular and neoplastic diseases, and expectations of the baby boomers include increased longevity as well as good quality of life. Consequently, the challenge of improving the effectiveness of OA treatments is of significant importance, particularly if the treatment may also reduce or halt progression of the disease.

The pharmacological treatment of OA includes the use of agents such as nonsteroidal anti-inflammatory drugs but also others, such as avocado/soybean unsaponifiables Expanscience™ (ASU; Laboratoires Expanscience, Courbevoie, France) [1], which are composed solely of the total fraction of unsaponifiables of avocado and soybean oils in proportions of one-third to two-thirds, respectively. ASU are a member of what are called 'slow-acting drugs for OA', which have been demonstrated to be effective in relieving OA symptoms [2].

Preclinical studies have shown that, *in vitro*, ASU have an inhibitory effect on IL-1β and stimulate collagen synthesis in articular chondrocytes [3]. In another *in vitro *model, ASU prevented – in part – the deleterious action of IL-1β on synovial cells and on rabbit articular chondrocytes [4]. They can also inhibit the stimulating action of IL-1β on stromelysin and collagenase and inhibit production of IL-6, IL-8 and prostaglandin E_2 _[5]. In addition, it was demonstrated that ASU could exert an anabolic effect by stimulating the expression of transforming growth factor (TGF)-β_1 _and plasminogen activator inhibitor-1 by articular chondrocytes [6]. Oral treatment with ASU in normal dogs was also shown to increase TGF-β_1 _and TGF-β_2 _levels in knee synovial fluid [7]. *In vivo*, ASU were found to reduce significantly the occurrence of lesions on cartilage in the postcontusive model of OA in rabbits [8] and to improve the subchondral bone structure in an ovine OA model induced by meniscectomy [9].

In addition to the above findings, and most interesting are the results from clinical trials that have shown a beneficial effect of ASU on clinical symptoms of knee and hip OA, with a carry-over effect that persists after termination of treatment [10-12].

The primary aim of the present study was to explore the effects of treatment with ASU on the development of early structural changes in an experimental OA dog model. The second objective was to identify the mechanisms by which the effects of ASU are exerted in this model. In brief, this study was designed to provide useful insight into the mode of action of ASU on the OA pathological process.

## Materials and methods

### Experimental group

Sixteen adult crossbred dogs (aged 2 to 3 years), each weighing 20 to 25 kg, were used in this study. They were housed in a large kennel in individual galvanized steel cages (1 m [width] × 1.75 m [length] × 2.4 m [height]), each separated by a panel. All cages were equipped with an automatic watering system. Dogs were selected after complete physical and musculoskeletal evaluations by a veterinarian. Haematological and biochemical analyses were conducted in the animals before their inclusion in the study. Surgical sectioning of the anterior cruciate ligament (ACL) of the right knee was performed on all dogs, as previously described [13] but with modifications. This model was created by sectioning of the ACL after joint capsule opening under general anaesthesia with pentobarbital sodium (25 mg/kg). All dogs received a multimodal and pre-emptive analgesic protocol based on opioid (fentanyl patch 75 μg [Janssen-Ortho, Markham, ON, Canada] and meperidine 4 mg/kg subcutaneous injection [Sandoz, Montreal, QC, Canada]) and intra-articular analgesia (marcaine 1 mg/kg [Hospira, Quebec, QC, Canada]). During the postoperative period, if needed, dogs were treated with fentanyl patch, oxymorphone (0.1 mg/kg SC; Sandoz) or meperidine SC, repeated as necessary. After surgery, the dogs were housed on a farm where they were free to exercise in a large enclosure. All dogs actively exercised in exterior runs (1.35 m [width] × 9.15 m [length]) for a 2-hour period, 5 days a week, under the supervision of an animal care technician.

The OA dogs were randomly assigned to two treatment groups, to which the animal care personnel were blinded. Dogs assigned to group 1 (n = 8) received placebo treatment (encapsulated methylcellulose) and those assigned to group 2 (n = 8) received ASU (Piascledine™; Expanscience, Courbevoie, France) orally once a day, every day including weekends, at a dosage of 10 mg/kg per day, which corresponds to twice the recommended daily dosage for the treatment of patients with knee or hip OA. The dosage was established based on the recent FDA guidelines [14]. Drug treatment was initiated immediately after surgery and continued until the dogs were euthanized 8 weeks later. The study protocol was approved by the institutional ethics committee and conducted in accordance with the Canadian Council on Animal Care guidelines.

### Macroscopic grading

Immediately after sacrifice, the right knee of each dog was placed on ice and dissected. Each knee was examined for gross morphological changes, as previously described, by two independent observers who were blinded to treatment group allocation [13].

The degree of osteophyte formation was graded by measuring the maximal width (mm) of the spurs on the medial and lateral femoral condyles using a digital caliper (Digimatic Caliper; Mitutoyo Corporation, Kawasaki, Japan). These two values, recorded for each dog, were considered separately for the purposes of statistical analysis.

The medial and lateral menisci of each knee were also scored macroscopically at the time of dissection as intact, fibrillated or torn, based on a method modified from that reported by Cook and coworkers [15].

The macroscopic lesion sizes at the cartilage surface were measured (in mm^2^) as previously described [13]. Overall scores were obtained for the femoral condyles and tibial plateaus separately by summing the score for each region recorded.

### Histological grading

Full thickness cartilage sections were removed from the weight-bearing lesional areas of the femoral condyles and tibial plateaus, allowing standardization of sampling as recommended by the OA Research Society International guidelines [15]. Histological evaluation was performed on sagittal sections of cartilage removed from each femoral condyle and tibial plateau specimen [16]. Specimens were dissected, fixed in TissuFix #2 (Laboratoires Gilles Chaput, Montreal, QC, Canada) and embedded in paraffin for histological evaluation. Serial sections (5 μm) were stained with Safranin-O. Two independent observers (CB and JC), who were blinded to treatment group allocation, graded the severity (consensus score) of the OA lesions in each cartilage section, which was divided into three subregions [15], on a scale of 0 to 29, modified from that reported by Sakakibara and colleagues [17]. This scale was used to evaluate the severity of OA lesions based on the loss of Safranin-O staining (scale 0 to 4), cellular changes (scale 0 to 12), structural changes (scale 0 to 10, where 0 = normal cartilage structure and 10 = complete disorganization) and pannus formation (scale 0 to 3). The final score (range 0 to 87) corresponds to the sum of the final scores for the three subregions of each specimen from the femoral condyle or tibial plateau.

Synovial membrane was removed and processed as described above for histological analysis. Samples were stained with haematoxylin-phloxine-saffron. The severity of synovitis was graded on a scale of 0 to 10 [13] by two blinded and independent observers (CB and JC, consensus score), who added the scores of histologic criteria: synovial cell hyperplasia (scale 0 to 2), villous hyperplasia (scale 0 to 3), and mononuclear (scale 0 to 4) and polymorphonuclear (scale 0 to 1) cell infiltration; 0 indicates normal structure.

### Subchondral bone and calcified cartilage histomorphometry

Specimens of full-thickness sections, which included the calcified cartilage and subchondral bone, were removed from the OA knee of all dogs and were placed in 70% ethanol and further decalcified with rapid bone decalcifier (RDO; Apex Engineering, Aurora, IL, USA). Specimens were embedded in paraffin for the purpose of histomorphometric analysis.

Sections (5 μm) of each specimen were subjected to hematoxylin/eosin staining. A Leitz Diaplan microscope (Leica Microsystems, Wetzlar, Germany) connected to a personal computer (Pentium IV based, using OSTEO II Image Analysis Software [Bioquant, Nashville, TN, USA]) was used to conduct bone histomorphometry analysis.

Subchondral bone histomorphometry was performed on three nonconsecutive sections of each specimen using our previously published method [18], modified from that of Matsui and coworkers [19]. The calcified cartilage/subchondral bone junction was used as the upper limit of each field. The depth was measured from the upper limit to 2,000 μm. Measurement of the bone surface (% of tissue surface) and trabecular thickness (μm) followed standard conventions, as previously described [18]. The measurement of the fields was then averaged for each section. Values for each section were considered separately for the purposes of statistical analysis.

Calcified cartilage histomorphometry was also done on three nonconsecutive sections of each specimen, as previously described [18]. From each section, three representative fields of 1,000 μm length (original magnification ×60) were selected. The tidemark/cartilage and calcified cartilage/bone junctions were used as upper and lower limits. The mean thickness (mm) of the calcified cartilage was calculated. The measurement made in the three fields was then averaged for each section and the value of each section was considered separately.

### Immunohistomorphometry

Cartilage specimens from the condyles and plateaus were processed for immunohistochemical analysis, as previously described [16,20], fixed in TissuFix #2 (Laboratoires Gilles Chaput) for 24 hours, and then embedded in paraffin. Serial sections (5 μm) of paraffin-embedded specimens were placed on Superfrost Plus slides (Fisher Scientific, Nepean, ON, Canada), de-paraffinized in toluene, rehydrated in a reverse graded series of ethanol and pre-incubated with 0.25 units/ml chondroitinase ABC (Sigma-Aldrich Canada, Oakville, ON, Canada) in phosphate-buffered saline (PBS; pH 8.0) for 60 minutes at 37°C. The specimens were subsequently washed in PBS, incubated in 0.3% Triton X-100 for 20 minutes, and then placed in 3% hydrogen peroxide/PBS for 15 minutes. Slides were further incubated with a blocking serum (Vectastain ABC kit; Vector Laboratories, Inc., Burlingame, CA, USA) for 60 minutes, after which they were blotted and then overlaid with the primary antibody against the following: inducible nitric oxide synthase (iNOS; 1/200, rabbit polyclonal; Santa Cruz Biotechnology Inc. [ref. #SC-650], Santa Cruz, CA, USA), matrix metalloproteinase (MMP)-1 (1/40 dilution, mouse monoclonal; Calbiochem ref. #444209; EMD Biosciences, Darmstadt, Germany), MMP-13 (1/6, goat polyclonal antibody; R&D Systems, Minneapolis, MN, USA), a disintegrin and metalloproteinase domain with thrombospondin motifs (ADAMTS)5 (1/50, rabbit polyclonal; Cedarlane [ref. #CL1-ADAMTS5], Hornby, ON, Canada) and ADAMTS4 (1/40, rabbit polyclonal; Cedarlane [ref. #CL1-ADAMTS4]) for 18 hours at 4°C in a humidified chamber.

Each slide was washed three times in PBS (pH 7.4), stained using the avidin-biotin complex method (Vectastain ABC kit), and incubated in the presence of the biotin-conjugated secondary antibody for 45 minutes at room temperature followed by the addition of the avidin-biotin-peroxidase complex for 45 minutes. All incubations were carried out in a humidified chamber at room temperature and the colour was developed with 3,3'-diaminobenzidine (DAKO Diagnostics Canada Inc., Mississauga, ON, Canada) containing hydrogen peroxide. The slides were counterstained with haematoxylin/eosin.

To determine the specificity of staining, three control procedures were employed in accordance with the same experimental protocol: omission of the primary antibody; substitution of the primary antibody with an autologous preimmune serum; and immunoadsorption with immunizing peptide for iNOS (Santa Cruz Biotechnology Inc.) and ADAMTS4 and ADAMTS5 (Cedarlane) or MMP-1 and MMP-13 recombinant protein (R&D Systems). As previously demonstrated, all controls exhibited only background staining (data not shown) [16]. Each section was examined under a light microscope (Leitz Orthoplan; Leica Inc., St. Laurent, QC, Canada) and photographed using a CoolSNAP cf Photometrics camera (Roper Scientific, Rochester, NY, USA).

The presence of the antigen in the cartilage was quantified using our previously reported method [13,16,21]. and estimated by determining the number of chondrocytes that stained positive throughout the entire thickness of the cartilage. Three sections from each femoral condyle and tibial plateau were examined, and each one was separately scored. The resulting data were integrated as a mean for each specimen. The cartilage was divided into six microscopic fields – three in the superficial zone and three in the deep zone (×40; Leica Inc.) – and the results were averaged for each zone. Before evaluation, it was ensured that an intact cartilage surface for each OA specimen could be detected and used as a marker for validation of the morphometric analysis. The superficial zone of cartilage corresponds to the superficial and the upper intermediate layers, and the deep zone to the lower intermediate and deep layers. The total number of chondrocytes and those staining positive in each zone for the specific antigen were determined. The final results were expressed as the percentage of chondrocytes staining positive for the antigen (cell score), with the maximum score being 100%. Each slide was subjected to two independent readers who were blinded to treatment group allocation. The final score was a consensus between the two observers (CB and JC).

For the purposes of statistical analysis, the data obtained from the femoral condyles and tibial plateaus were considered separately. iNOS, MMP-1, ADAMTS4 and ADAMTS5 were quantified in the superficial zone of cartilage because the staining for these antigens was found to be negligible in the deep zone, whereas MMP-13 was quantified in the deep zone, which is its preferential zone of expression [22].

### Statistical analysis

Macroscopic and histologic data are expressed as mean ± standard error of the mean. Immunohistochemical and histomorphometric results are expressed as the median (range). Statistical analysis, unless otherwise specified, was performed using the Mann-Whitney U-test or Student's unpaired *t*-test. *P *values of less than or equal to 0.05 were considered statistically significant.

## Results

### Macroscopic findings

#### Osteophytes

The incidence and size of the osteophytes were similar for both groups, with a mean size of 4.77 ± 0.32 mm (mean ± standard error of the mean) for the placebo group and 5.24 ± 0.30 mm for the ASU-treated group.

#### Meniscus

The macroscopic evaluation of the medial and lateral menisci revealed no difference between placebo and ASU-treated groups. In the medial compartment, two of the eight dogs in the placebo group exhibited a meniscal tear, as compared with three in the ASU-treated group; five of the eight dogs in the placebo group had a fibrillated meniscus versus four in the ASU-treated group; and one dog in each group had an intact medial meniscus. In the lateral compartment, six of the eight dogs in the placebo group had an intact lateral meniscus versus seven in the ASU-treated group, and two menisci were fibrillated in the placebo group versus one in the ASU-treated group.

#### Cartilage

**Figure 1:**
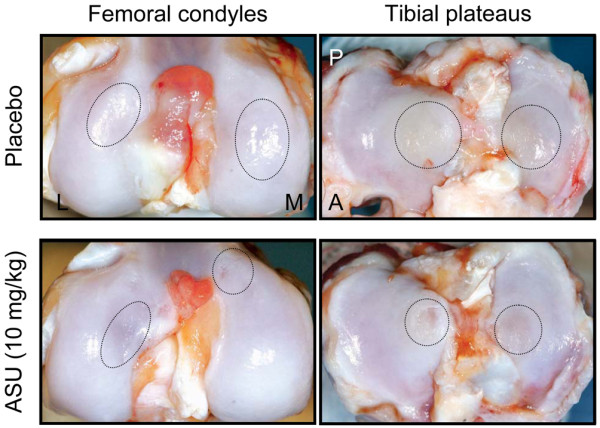
Macroscopic appearance of osteoarthritic articular cartilage from femoral condyles and tibial plateaus. Representative pictures of placebo-treated and ASU-treated dogs at 8 weeks after surgery, showing erosion and pitting (circled). A, anterior; L, lateral; M, medial; P, posterior.

The severity (surface) of the lesions on the tibial plateaus was significantly decreased in the ASU-treated dogs (50.5 ± 4.0 mm^2^) compared with the placebo-treated group (67.1 ± 6.1 mm^2^; *P *= 0.04; Figure 1). The severity (surface) of the lesions on the femoral condyles in the ASU-treated dogs (29.3 ± 5.7 mm^2^) was less than that in the placebo-treated dogs (38.1 ± 5.5 mm^2^; Figure 1).

### Histological findings

#### Cartilage

**Figure 2:**
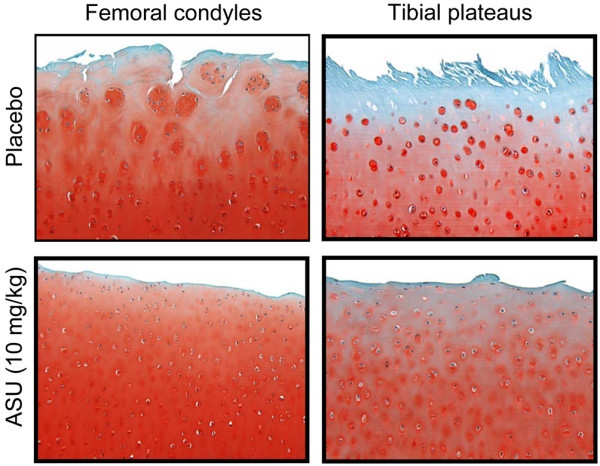
Histology of osteoarthritic articular cartilage from the femoral condyles and tibial plateaus. Representative sections of placebo-treated and ASU-treated dogs 8 weeks post-surgery (original magnification ×100). Sections were stained with Safranin-O. ASU, avocado/soybean unsaponifiables.

The cartilage specimens from the dogs treated with placebo exhibited modifications that are typical of OA. The total histological scores for the severity of cartilage lesions on the femoral condyles and the tibial plateaus were significantly decreased in the ASU-treated dogs compared with those treated with placebo (*P *< 0.0001 for femoral condyles and *P *= 0.0002 for tibial plateaus; Figure 2 and Table 1). On the femoral condyles and tibial plateaus, the scores of all parameters were significantly decreased in the ASU-treated group compared with the placebo-treated group, with the exception of the Safranin-O staining and the pannus on the plateaus (Table 1).

**Table 1:** Histological score of femoral condyles and tibial plateaus of placebo and ASU-treated dogs

	Treatment group	Structure (0 to 30)^a^	Tangential (0 to 6)^a^	Transitional (0 to 30)^a^	Safranin-O staining (0 to 12)^a^	Pannus (0 to 9)^a^	Total (0 to 87)^a^
Femoral condyles	Placebo	20.50 ± 0.92	5.75 ± 0.19	15.63 ± 0.70	4.69 ± 0.33	0.88 ± 0.24	47.55 ± 1.74
	ASU (10 mg/kg)	11.44 ± 0.90 (*P *< 0.0001)	3.81 ± 0.25 (*P *< 0.0001)	12.88 ± 0.42 (*P *= 0.006)	3.69 ± 0.24 (*P *= 0.03)	0.19 ± 0.10 (*P *= 0.004)	32.31 ± 1.64 (*P *< 0.0001)
Tibial plateaus	Placebo	20.69 ± 0.64	5.75 ± 0.14	16.88 ± 0.79	4.75 ± 0.40	1.75 ± 0.34	49.81 ± 1.86
	ASU (10 mg/kg)	13.25 ± 1.52 (*P *< 0.0001)	4.19 ± 0.28 (*P *= 0.0002)	13.06 ± 0.55 (*P *= 0.001)	4.25 ± 0.31	1.62 ± 0.36	36.38 ± 2.07 (*P *= 0.0002)

The score was determined as described in the Materials and methods section. The final score corresponds to the sum of the scores obtained for the three subregions. Data are expressed as mean ± standard error of the mean and were analyzed using Mann-Whitney two-tailed U-test. Comparison was performed with the autologous placebo and *P *= 0.05 is considered statistically significant. ^a^The values given in parentheses indicates the range for each measure. ASU, avocado/soybean unsaponifiables.

#### Synovial membrane

The synovial membranes from the placebo-treated dogs exhibited hyperplasia of the lining cells, villous hyperplasia and mononuclear cellular infiltration. ASU treatment induced a slight reduction in the total histological score (7.13 ± 0.48 for the placebo group and 5.63 ± 0.56 for the ASU-treated group). The histological score was identical in both groups for all of the criteria except for cellular infiltration. Indeed, ASU induced a significant decrease in cellular infiltration (3.00 ± 0.19 for the placebo group and 1.50 ± 0.50 [*P *= 0.04] for the ASU-treated group).

### Bone histomorphometric analysis

**Figure 3:**
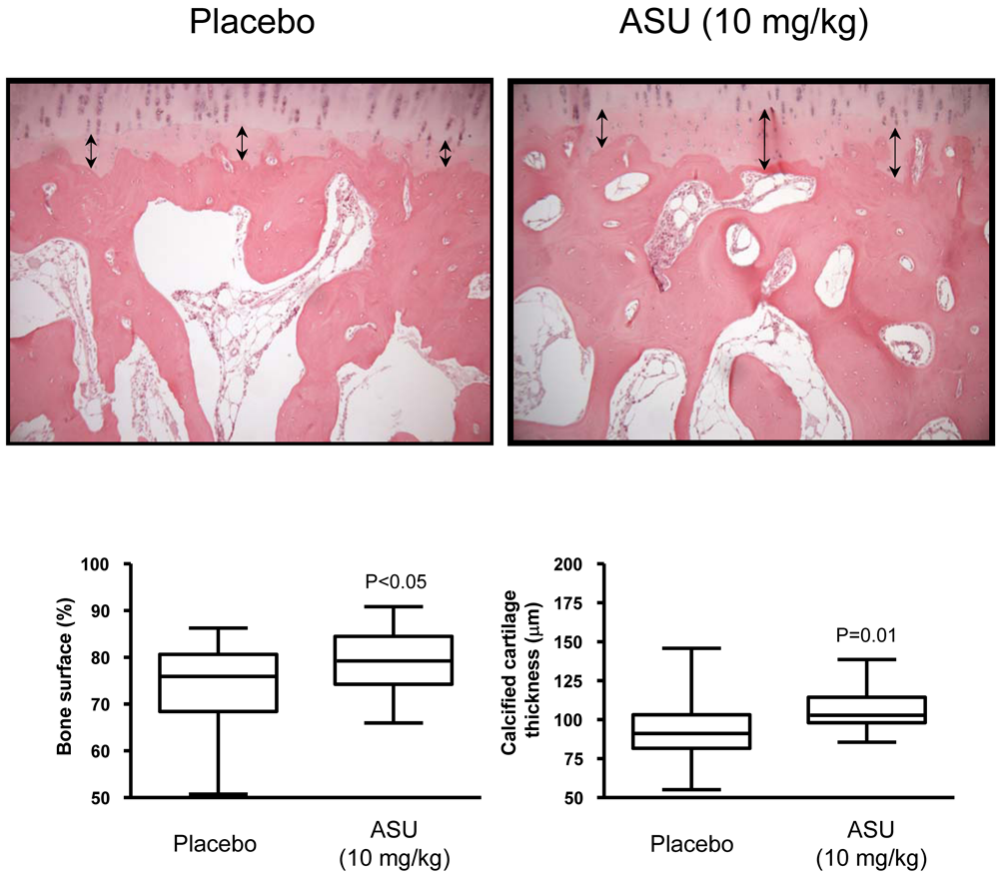
Subchondral bone and calcified cartilage. Representative sections and data of placebo-treated and ASU-treated dogs 8 weeks post-surgery (original magnification ×100). Sections were stained with haematoxylin/eosin. Data for bone volume and calcified cartilage thickness are represented as box plot and were analyzed using Student's unpaired *t*-test. *P *≤ 0.05 is considered significant. ASU, avocado/soybean unsaponifiables.

Bone surface was 75.9% (50.8% to 86.2%; median [range]) of the tissue surface in the placebo group compared with 79.3% (66.0% to 90.8%; *P *< 0.05) in the ASU-treated group (Figure 3). Trabecular thickness did not differ between the ASU (132.3 μm [102.8 μm to 200.5 μm]) and placebo (129.1 μm [90.0 μm to 156.0 μm]) groups.

The calcified cartilage thickness was significantly greater in the ASU group (102.9 μm [85.5 μm to 138.7 μm]) than in the placebo group (91.2 μm [55.2 μm to 145.9 μm]; *P *= 0.01).

### Immunohistomorphometric findings

**Figure 4:**
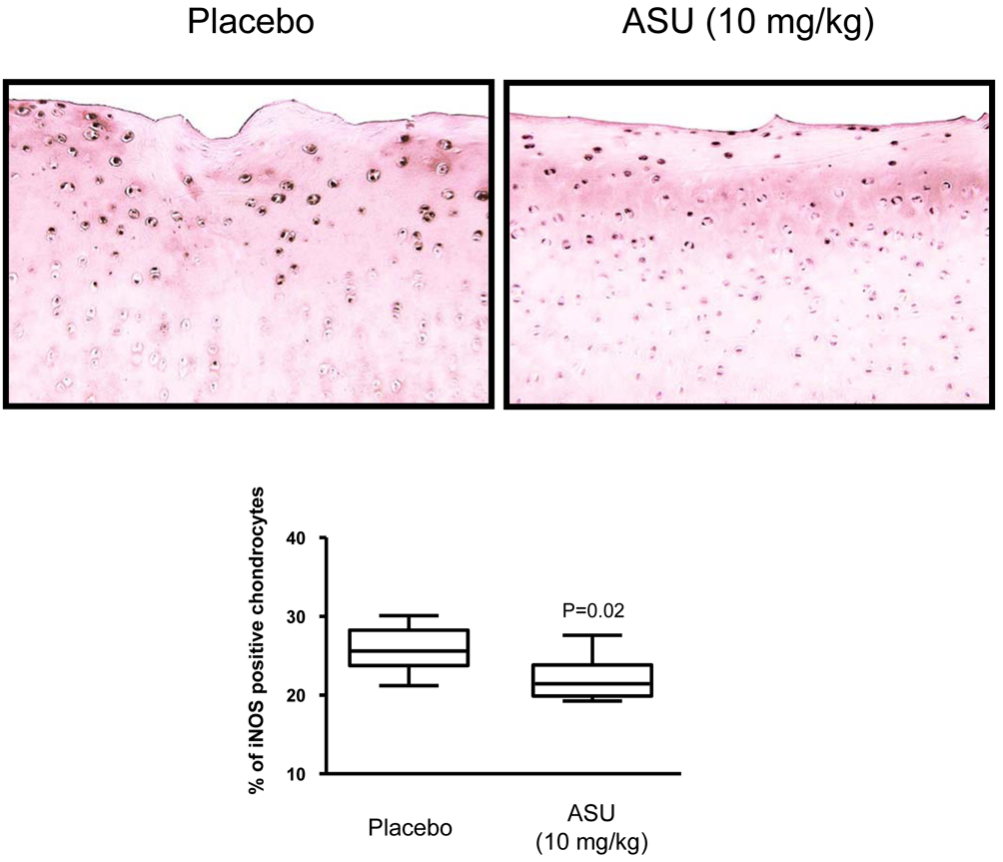
iNOS immunostaining. Representative sections and data of the superficial zone of articular cartilage from placebo-treated and ASU-treated dogs (original magnification ×100). Morphometric analysis of iNOS immunostaining data are represented as box plot and were analyzed using Mann-Whitney two-tailed U-test. *P *≤ 0.05 is considered statistically significant. ASU, avocado/soybean unsaponifiables; iNOS, inducible nitric oxide synthase.

Chondrocytes staining positive for iNOS were found preferentially located in the superficial zone of the OA cartilage. The percentage of positive cells was found to be significantly decreased in the ASU-treated group (21.4% [19.3% to 27.6%]) compared with the placebo-treated group (25.6% [21.2% to 30.1%]; *P *= 0.02; Figure 4).

**Figure 5:**
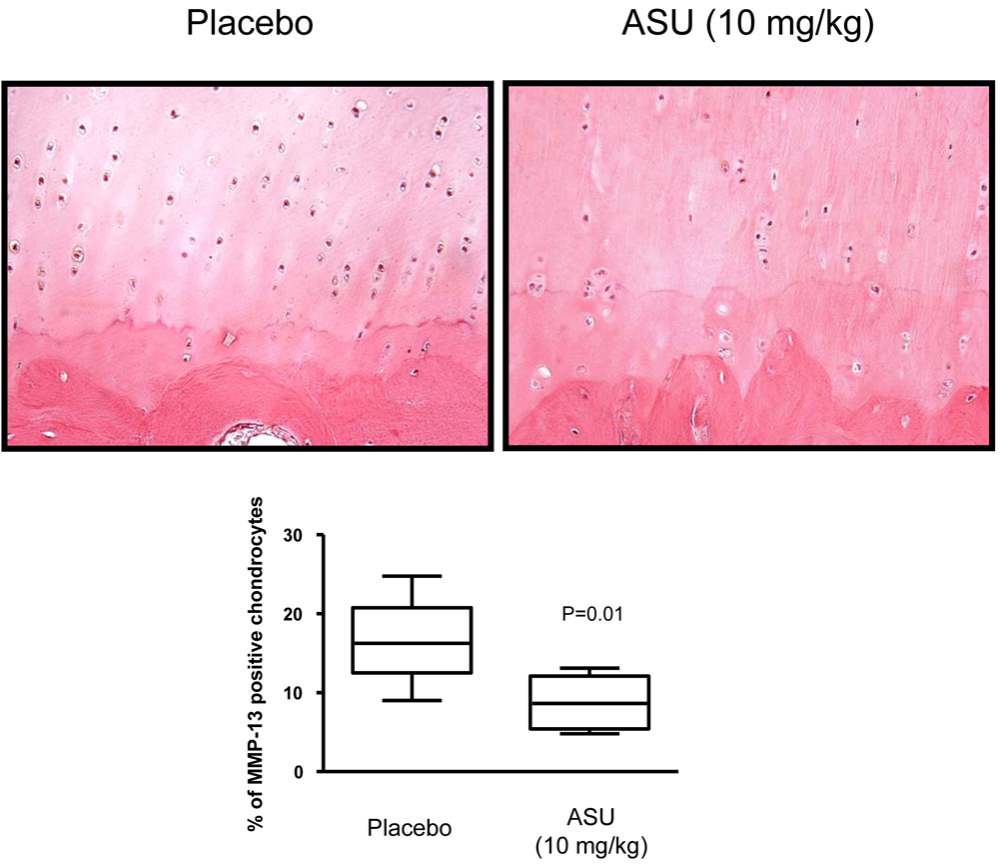
MMP-13 immunostaining. Representative sections and data of the deep zone of articular cartilage from placebo-treated and ASU-treated dogs (original magnification ×250). Morphometric analysis of MMP-13 immunostaining data are represented as box plot and were analyzed using Mann-Whitney two-tailed U-test. *P *≤ 0.05 is considered statistically significant. ASU, avocado/soybean unsaponifiables; MMP, matrix metalloproteinase.

In contrast to iNOS, MMP-13 was detected preferentially in the deep zone of OA cartilage. Dogs treated with ASU exhibited a significant reduction in the level of MMP-13 (8.6% [4.8% to 13.10%]) compared with the placebo-treated group (16.3% [9.0% to 24.8%]; *P *= 0.01; Figure 5).

The levels of other mediators were also studied and found to be similar in the two groups: MMP-1, 28.0% (23.2% to 30.9%) of chondrocytes in the placebo group versus 29.6% (27.8% to 30.7%) in the ASU-treated group; ADAMTS4, 23.9% (22.0% to 28.2%) versus 26.9% (22.9% to 28.9%); and ADAMTS5, 24.6% (20.7% to 29.8%) versus 26.05% (17.9% to 29.5%).

## Discussion

In the experimental dog OA model induced by the sectioning of the ACL, treatment with ASU can reduce the development of early OA cartilage and subchondral bone lesions. The histological findings were informative with respect to the effects of ASU on the OA cartilage structural changes. In fact, dogs treated with ASU exhibited a significant decrease in indicators of cartilage matrix damage, such as the structural changes indicative of collagen network breakdown and Safranin-O staining, which indicates aggrecan degradation. In addition, treatment with ASU was found to reduce chondrocyte hyperplasia and cloning. These findings are in accordance with those of Cake and coworkers [9] in an ovine meniscectomy model of OA.

Many proteases have been shown to play major roles in the catabolism of OA cartilage. For instance, MMP-13 has been demonstrated to play a predominant role in the degradation of collagen type II in OA cartilage [23], whereas ADAMTS4 and ADAMTS5 are believed to be key proteases in the degradation of aggrecans [24,25]. In the ACL dog model, inhibition of the synthesis of these enzymes by treatment with different drugs, such as pioglitazone (a peroxisome proliferator-activated receptor-γ agonist [26]) and licofelone (a dual inhibitor of 5-lipoxygenase and cyclo-oxygenase [27]), has been found to be associated with a reduction in the development of cartilage lesions. These enzymes, by cleaving the triple helix of collagen type II and core protein of the aggrecan respectively, induce major irreversible damage to the cartilage matrix structure. In so doing, they can modify the biophysical properties of cartilage and reduce its resilience to the abnormal biomechanical forces present in OA.

In the present study, treatment with ASU reduced the level of MMP-13 synthesis in the deep zone of cartilage. These findings are in accordance with a previous study in which ASU were demonstrated to reduce MMP-13 mRNA in murine chondrocytes in monolayer culture under stimulation with IL-1β [28]. MMP-13 expression is increased in tissue that is in need of repair or remodelling, as in OA. MMP-13 was previously shown to be preferentially increased in the deep zone of cartilage [22,29]. and was described as a major catabolic factor in that zone as well as in OA lesional areas [30,31]. Therefore, our finding of the effect of ASU treatment on reduction in MMP-13 synthesis could explain the prevention of OA cartilage lesion development as well as the protective effect of ASU on the erosion of the calcified cartilage [19]. On the other hand, ASU were found to have no effect on the level of synthesis of other proteases involved in cartilage matrix degradation, such as MMP-1, ADAMTS4 and ADAMTS5; these enzymes are believed to be predominantly involved in matrix degradation in the more superficial zones of cartilage. These results support the hypothesis that the reduction in collagen degradation in the deep zone of cartilage could reduce the development of OA lesions. Alternatively, the absence of effect on the above proteases may explain the mild to moderate effect that ASU had on the reduction in OA cartilage lesions.

MMP-13 is known to be involved in subchondral bone remodelling and resorption of calcified cartilage in OA [18]. As previously demonstrated by Cake and coworkers [9], ASU treatment was able to protect the remodelling of subchondral bone in an ovine OA model. Moreover, studies suggest that drugs that can reduce MMP-13 synthesis in cartilage and bone and prevent subchondral bone resorption in the dog ACL model could exert a disease-modifying effect in knee OA patients [18,27]. Our findings demonstrate that ASU could reduce OA subchondral bone remodelling and resorption, which leads to osteopenia, a phenomenon well documented in the ACL dog OA model that occurs during the first few months after surgery [32,33]. The bone volume and calcified cartilage thickness in the ASU-treated group were greater than those found in the placebo group and close to the morphometric values found in normal dogs [18]. These findings indicate that the ASU treatment reduced the loss of subchondral bone. Moreover, ASU treatment was able to maintain subchondral bone and calcified cartilage structure close to normal values.

ASU treatment prevented the loss, but it did not increase bone surface or calcified cartilage thickness over the values found in normal dogs [18]. These data also support the concept that the subchondral bone is the site of morphological changes that are part of the OA disease process [5,8,10-12,18,34], and provide further evidence in favour of the concept that therapeutic intervention to reduce these changes may also prevent the development of cartilage lesions. This latter hypothesis is further supported by a number of published studies showing that, in ACL OA models, treatment with calcitonin [33] and alendronate [35] reduced bone resorption as well as cartilage degeneration. In this context, the study conducted by Henrotin and coworkers [36], in which ASU reversed the inhibition of aggrecan and collagen synthesis in OA chondrocyte/osteoblast co-culture, is supportive of the existence and key role played by the crosstalk between cartilage and subchondral bone in OA pathophysiology.

In contrast to normal cartilage, OA cartilage produces an excess amount of nitric oxide (NO) upon iNOS (the enzyme responsible for NO production) stimulation by cytokines [37,38]. High levels of nitrite/nitrate have also been found in the synovial fluid and serum of arthritis patients [39] as well as in synovial tissue from OA patients [40,41]. It has been hypothesized that NO contributes to the development of arthritic lesions [42-44] by inhibiting the synthesis of cartilage matrix macromolecules [45-48] and by inducing chondrocyte death [49,50], which could further contribute to the reduction in extracellular matrix in OA. NO was also shown to reduce the synthesis of the IL-1 receptor antagonist in chondrocytes [38], a process possibly responsible for the enhanced IL-1β effect on these cells. Moreover, the diffusion of NO from the superficial layer of cartilage to the deeper zone may also have contributed to increasing the level of MMP-13 synthesis at that level [51]. An *in vivo *study with *N*-iminoethyl-L-lysine, a potent and selective iNOS inhibitor [52], demonstrated its therapeutic effectiveness in reducing the progression of experimental OA in the ACL dog model [53]. The same study also demonstrated that iNOS inhibition reduced the synovial inflammation, a finding that is well in agreement with those of the present study. Moreover, ASU exhibited an inhibitory effect on iNOS, and therefore on NO production, which may provide an explanation for the protective effect of ASU. These results are in accordance with those of a study in human OA chondrocytes previously published by Henrotin and coworkers [54].

The present study has limitations largely imposed by the study design. One such limitation is the duration of the study (8 weeks). A longer study would provide more information on the potential effects of ASU against the long-term development of OA. Moreover, the study design involved prophylactic use of the drug and the conclusions drawn might have been influenced by this treatment schedule. A further study in which therapeutic administration is employed would be informative and complementary to the present one. The mechanisms of action of ASU, especially their global effect on catabolic/anabolic factors, needs further investigation in order to reach a better understanding of the disease pathways that are modified by this treatment.

## Conclusions

The present findings indicate that the protective effect of ASU on OA structural changes could be mediated by a reduction in cartilage catabolism and in subchondral bone remodelling, which may be due, at least in part, to their inhibitory effects on iNOS and MMP-13.

## Abbreviations

ACL: anterior cruciate ligament; ASU: avocado/soybean unsaponifiables; iNOS: inducible nitric oxide synthase; MMP: matrix metalloproteinase; NO: nitric oxide; OA: osteoarthritis; PBS: phosphate-buffered saline; TGF: transforming growth factor.

## Competing interests

This study was supported in part by a grant from Laboratoires Expanscience (10, avenue de l'Arche 92419 Courbevoie Cedex, France). JM-P and JPP are consultants for Laboratoires Expanscience. PM, GBG and CB are employees of Laboratoires Expanscience.

## Authors' contributions

JPP, JM-P, PM, GBG and CB participated in the study design. CB, JC and JPP participated in the acquisition of data. CB, JM-P, JC and JPP participated in the analysis and interpretation of data. CB, JM-P and JPP prepared the manuscript. CB, JM-P and JC participated in the statistical analysis.
